# The *TERT* promoter SNP rs2853669 decreases E2F1 transcription factor binding and increases mortality and recurrence risks in liver cancer

**DOI:** 10.18632/oncotarget.6331

**Published:** 2015-11-09

**Authors:** Eunkyong Ko, Hyun-Wook Seo, Eun Sun Jung, Baek-hui Kim, Guhung Jung

**Affiliations:** ^1^ Department of Biological Sciences, College of Natural Sciences, Seoul National University, Gwanak-gu, Seoul, 151-747, South Korea; ^2^ Department of Pathology, Seoul St. Mary's Hospital, The Catholic University of Korea, Seocho-Gu, Seoul, 133-782, South Korea; ^3^ Department of Pathology, Korea University Guro Hospital, Korea University College of Medicine, Seoul, 152-703, South Korea

**Keywords:** single-nucleotide polymorphism at telomerase reverse transcriptase (TERT) promoter, TERT promoter mutation, risk of hepatocellular carcinoma (HCC)-related mortality and recurrence, mechanism for regulation of SNP-dependent TERT promoter activity, A TERT transcription repressor

## Abstract

A common single-nucleotide polymorphism in the telomerase reverse transcriptase (*TERT*) promoter, rs2853669 influences patient survival rates and the risk of developing cancer. Recently, several lines of evidence suggest that the rs2853669 suppresses *TERT* promoter mutation-mediated *TERT* expression levels and cancer mortality as well as recurrence rates. However, no reports are available on the impact of rs2853669 on *TERT* expression in hepatocellular carcinoma (HCC) and its association with patient survival. Here, we found that HCC-related overall and recurrence-free survival rates were not associated with *TERT* promoter mutation individually, but rs2853669 and the *TERT* promoter mutation in combination were associated with poor survival rates. *TERT* mRNA expression and telomere fluorescence levels were greater in patients with HCC who had both the combination. The combination caused *TERT* promoter methylation through regulating the binding of DNA methyltransferase 1 and histone deacetylase 1 to the *TERT* promoter in HCC cell lines. The *TERT* expression level was significantly higher in HCC tumor with a methylated promoter than in that with an unmethylated promoter. In conclusion, we demonstrate a substantial role for the rs2853669 in HCC with *TERT* promoter mutation, which suggests that the combination of the rs2853669 and the mutation indicate poor prognoses in liver cancer.

## INTRODUCTION

Telomerase reverse transcriptase (TERT) is the catalytic subunit of telomerase [1], which is an essential enzyme for elongating telomeres at the end of chromosomes [2]. *TERT* expression levels are highly associated with cancer risk in various human cancers [1–3]. Cancer risk is associated with common single-nucleotide polymorphisms (SNPs), including the *TERT* gene variant, rs2853669 (−245T > C) [3]. Although rs2853669 increases lung cancer risk [4], it contributes to a lower breast cancer risk [3]. In studies of hepatocellular carcinoma (HCC), which accounts for more than 85% of liver cancers [5], no evidence has been reported on the significant association between the rs2853669 variant and HCC risk [6].

Telomerase activation is responsible for circumventing cellular senescence or cell death caused by telomere shortening in cells [7–9]. *TERT* expression, which is necessary for telomerase activity, is greater in various human tumors than in normal organs [2]. Recently, −124C > T and −146C > T somatic mutations at the *TERT* promoter were discovered in melanoma; a reporter assay showed that these mutations increased *TERT* transcription activity by creating a binding motif for transcription factor ETS2 in multiple cell lines [10–12]. These −124C > T and −146C > T somatic mutations are present in various human tumors, including HCC [1]. However, a number of previous reports disagree on whether *TERT* promoter mutations are responsible for elevated TERT expression or low patient survival rate in a variety of cancers, including, but not limited to, cutaneous melanoma [13, 14], thyroid cancer [15], bladder cancer [16–18], and HCC [19, 20]. Although *TERT* expression is greater in cirrhotic preneoplastic lesions (a HCC precursor) with the *TERT* promoter mutations compared with that of lesions that do not include the mutations [19], it is yet unclear whether somatic mutations at the *TERT* promoter affect the *TERT* expression levels or patient survival rates in HCC [19, 20].

Several lines of research suggest that rs2853669 suppresses the *TERT* promoter mutation-mediated *TERT* expression regulation and mortality or recurrence rates for bladder cancer [17], gliomas [21, 22], and renal cell cancer [23]; however, the mechanism by which *TERT* transcription is regulated remains unknown. So far, no studies have considered effect of rs2853669 combined with somatic mutations at the *TERT* promoter on liver cancer. Here, we aim to discover a novel role for the rs2853669 variant and a mechanism for regulating rs2853669 variant-dependent *TERT* promoter activity in liver cancer.

## RESULTS

### The combination of the rs2853669 variant and the TERT promoter mutation increases mortality and cancer recurrence rates in HCC patients

**Figure 1:**
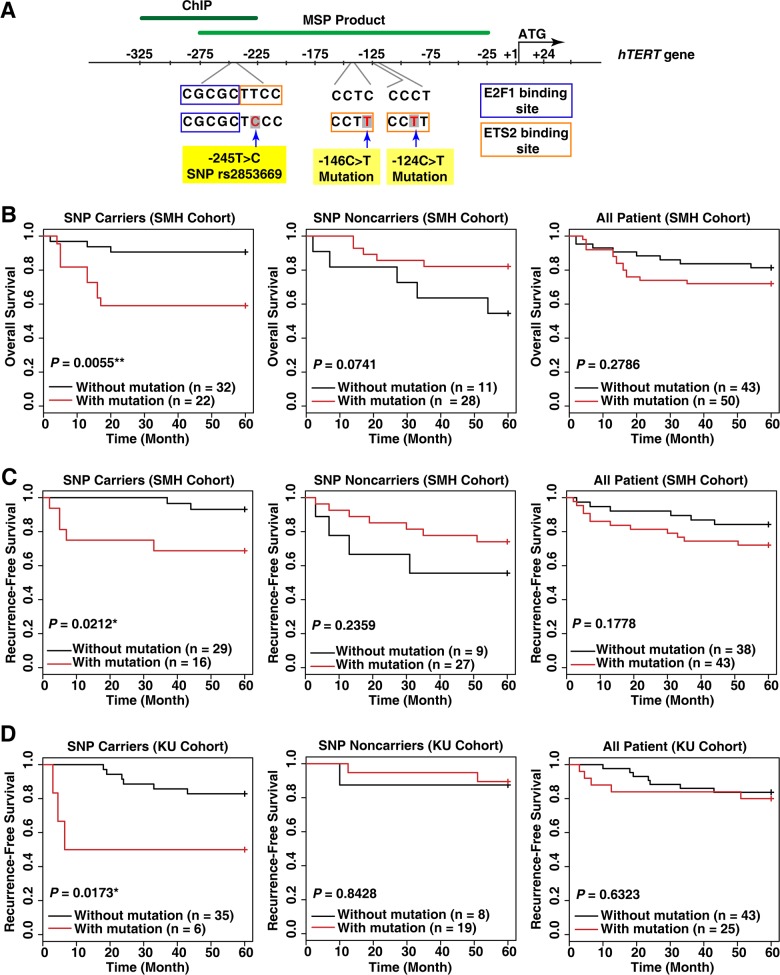
The variant rs2853669 at the *TERT* promoter is associated with a high risk of death and cancer recurrence in hepatocellular carcinoma (HCC) patients with a *TERT* promoter mutation **A.** A representative map describing the SNP rs2853669 (−245T > C), −146C > T mutation, and −124C > T mutation in the human *TERT* gene. ChIP, Chromatin immunoprecipitation; MSP product, methylation specific PCR product. **B.** Kaplan–Meier analysis of differences in overall survival and **C, D.** recurrence-free survival based on the presence of the rs2853669 variant and *TERT* promoter mutation status. HCC patients included the SMH cohort (B, C) and KU cohort (D) *P < 0.05; **P < 0.01.

To determine the combined effect of the rs2853669 (−245T > C) and *TERT* promoter mutation (−124C > T or −146C > T) on liver cancer survival rates, we first analyzed the overall survival rates of a Korean HCC patient cohort (SMH cohort, n = 93; Figure 1A, 1B). The combination was correlated with a low overall survival rate (Log-rank test, *P* = 0.0055; Figure 1B) and a high significant risk of HCC-related death, as evidenced by the hazard ratio of 5.259 (95% CI = 1.42–19.48, *P* = 0.013; Supplementary Table 1). We did not observe an association between the overall survival rate and the *TERT* promoter mutation in HCC patients lacking rs2853669 (Figure 1B). Moreover, no significant difference in the survival rates was observed among the HCC patients with or without the *TERT* promoter mutation (Figure 1B), which is consistent with previous reports [19, 20]. Therefore, the mutation alone does not lead to high mortality rates in HCC patients while we concluded that the *TERT* promoter mutation and rs2853669, when in combination, does.

Next, we examined whether rs2853669 and the *TERT* promoter mutation combination influenced the risk of HCC recurrence in two independent Korean HCC patient cohorts (n = 93 for the SMH cohort; n = 72 for the KU cohort). The data show that rs2853669 is associated with poor recurrence-free survival rates and a significant risk of HCC recurrence in patients with the *TERT* promoter mutation (Log-rank test, *P* = 0.02119; hazard ratio = 5.5611, 95% CI = 1.076–28.75, *P* = 0.0406 for the SMH cohort; and Log-rank test, *P* = 0.0173; hazard ratio = 4.6639, 95% CI = 1.157–18.8, *P* = 0.0304 for the KU cohort; Figure 1C, 1D and Supplementary Table 1). In accordance with the results of the overall survival analysis (Figure 1B), analysis of the recurrence-free survival rates revealed that in patients without the rs2853669 or in all patients (with and without the SNP), there was no significant difference in the recurrence rate in response to the *TERT* promoter mutation (Figure 1C, 1D). Furthermore, both the overall survival and recurrence-free survival rates did not differ significantly between the patients with and without the rs2853669 alone (Supplementary Figure 1). Overall, only the combination of the rs2853669 and the *TERT* promoter mutation contributed to a high risk of HCC recurrence.

### The combination of the rs2853669 variant and the TERT promoter mutation increases TERT transcription activity in HCC cell lines and HCC tumors

**Figure 2:**
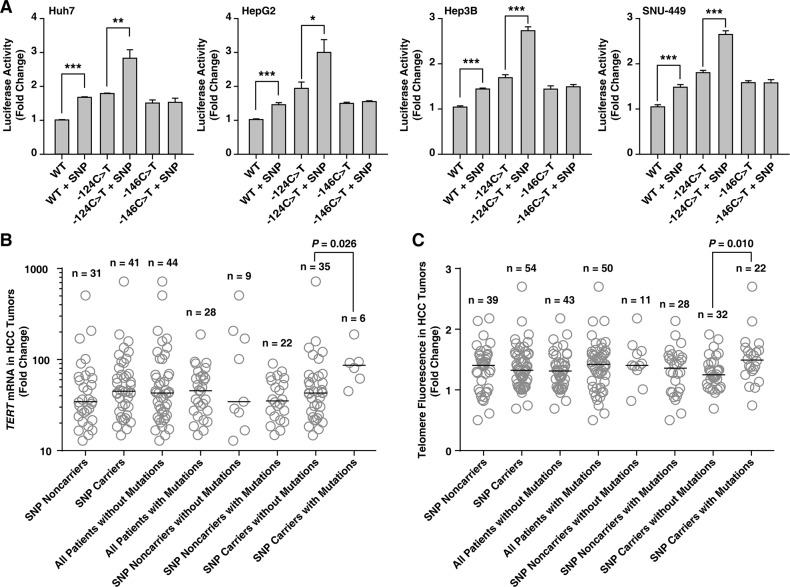
The variant rs2853669 at the *TERT* promoter is associated with an increased *TERT* promoter activity in HCC cell lines, and a high *TERT* mRNA expression level and long telomere lengths in HCC tumors **A–C.** Quantification of *TERT* promoter activity in hepatocellular carcinoma (HCC) cell lines (A), *TERT* expression (B) and telomere fluorescence levels (C) in HCC tumor tissues based on the presence of the variant rs2853669 (single-nucleotide polymorphism (SNP)) only, the mutation only (−124C > T or −146C > T), and a combination of both (−124C > T + SNP or −146C > T + SNP). The data in *A* are presented as the means ± SEM (n = 4). The horizontal bar in *B* and *C* show the median. *P < 0.05, **P < 0.01, and ***P < 0.001.

The luciferase promoter activity of the *TERT* reporter vector with rs2853669 (−245T > C) was increased in all four HCC cell lines examined (*t*-test, *P* < 0.001 for all cell lines; Figure 2A). Furthermore, the luciferase promoter activity of the *TERT* reporter vector with both rs2853669 and −124C > T was significantly greater than that of vectors with the −124C > T mutation only (*t*-test, *P* = 0.002 for Huh7, *P* = 0.012 for HepG2, *P* < 0.001 for Hep3B, and *P* < 0.001 for SNU-449; Figure 2A), which indicates a marked impact by the combination on *TERT* expression up-regulation.

Lastly, we examined whether the *TERT* mRNA expression level positively associate with the rs2853669 and the mutation combination. The *TERT* mRNA expression level was greater in HCC tumors with the combination than in tumors with rs2853669 only (Mann–Whitney test, *P* = 0.026; Figure 2B). Furthermore, HCC tumors with the combination had longer telomeres, which correlates with high *TERT* mRNA levels in HCC [24], than tumors with the rs2853669 polymorphism alone (Mann–Whitney test, *P* = 0.010; Figure 2C and Supplementary Figure 2). These data show rs2853669 variant and the *TERT* promoter mutation combination is positively associated with HCC tumor *TERT* mRNA expression levels and telomere lengths.

### Inhibiting E2F1 binding to the TERT promoter increases TERT transcription levels in Huh7 cells without the rs2853669 variant

**Figure 3:**
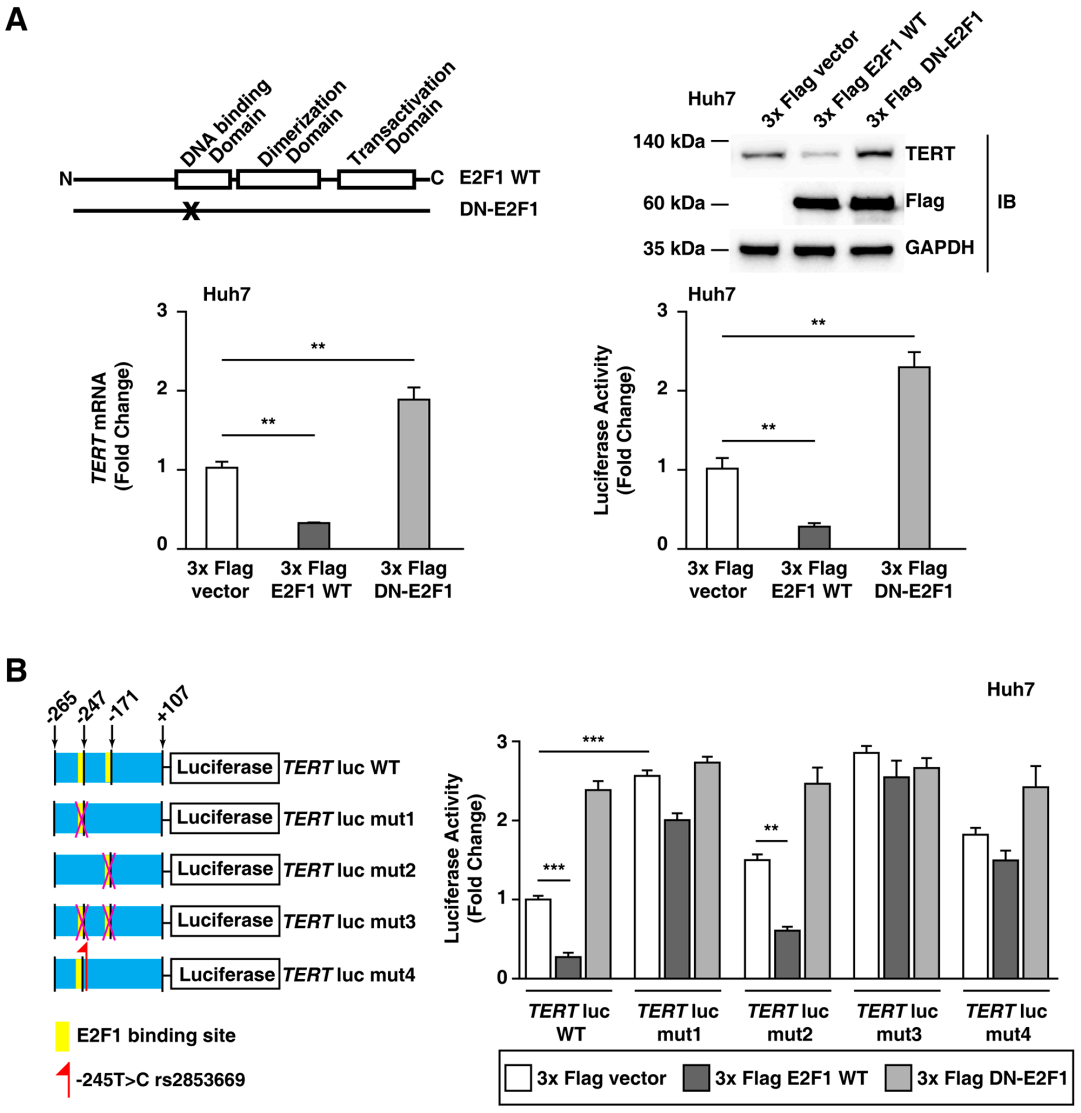
E2F1 is a *TERT* transcriptional repressor in Huh7 cells without rs2853669 **A.** Domain structure of wild-type E2F1 (E2F1 WT) and dominant-negative E2F1 (DN-E2F1); the immunoblot assay, qPCR, and the luciferase assay using a 3x Flag pCMV-10 empty vector, 3x Flag E2F1, and 3x Flag DN-E2F1. DN-E2F1 is mutated in the DNA binding domain. **B.** Luciferase assay using the WT *TERT* promoter (*TERT*-WT-luc), *TERT* promoter with a mutated E2F1-binding site (−247 bp upstream of ATG) (*TERT*-mut1-luc), *TERT* promoter with a mutated E2F1-binding site (−171 bp upstream of ATG) (*TERT*-mut2-luc), *TERT* promoter with mutated E2F1-binding sites (−247 bp upstream and −171 bp upstream of ATG) (*TERT*-mut3-luc), and *TERT* promoter with rs2853669 (−245 bp upstream of ATG) (*TERT*-mut4-luc). The data are shown as the mean ± SEM, **P < 0.01, *** *P* < 0.005.

We further investigated the mechanism underlying the role of the rs2853669 in the *TERT* transcription activation as observed in the HCC tumors with the rs2853669 variant and the mutation combination. The rs2853669 variant site is close to (2 bp downstream) the binding site of E2F transcription factor 1 (E2F1) (Figure 1A). Therefore, we evaluated whether the rs2853669 variant can disrupt a preexisting E2F1 binding site at the *TERT* promoter and inhibit the E2F1 function as a *TERT* transcription repressor. First, we tested whether the E2F1 protein was involved in the lower *TERT* gene expression levels in Huh7 cells, which are HCC cells without the rs2853669 variant (Supplementary Figure 3). As a result, the E2F1 wild-type (WT) overexpression decreased the *TERT* mRNA and TERT protein expression levels as well as the *TERT* gene promoter activity in Huh7 cells (Figure 3A). In contrast, E2F1-Eco132 ectopic expression, which is due to a dominant-negative E2F1 mutant [25], increased the *TERT* mRNA, protein expression levels, and *TERT* promoter activity (Figure 3A), suggesting that E2F1 is a *TERT* transcriptional repressor in Huh7 cells.

We confirmed whether the effect of the rs2853669 variant (−245T > C) on *TERT* promoter activity was associated with the function of E2F1 as a transcriptional repressor. A previous study showed that the two E2F1 binding sites at −247 bp and −171 bp relative to ATG can regulate *TERT* promoter activity [26]. As demonstrated by the luciferase assay, the E2F1-binding site mutation at −247 bp relative to ATG (*TERT*-mut1-luc) compared to the E2F1-binding site mutation at −171 bp relative to ATG (*TERT*-mut2-luc) had a greater impact on the *TERT* promoter activity (Figure 3B). In addition, *TERT*-mut2-luc luciferase activity decreased after E2F1 overexpression (*P* < 0.01); however, *TERT*-mut1-luc luciferase activity was not affected by E2F1 overexpression (Figure 3B). Moreover, a subsequent luciferase assay demonstrated that the increased E2F1 level did not influence the luciferase activity of the *TERT* promoter with the rs2853669 variant (*TERT*-mut4-luc), similar to that demonstrated for the *TERT* promoter with a mutation at the E2F1-binding site (*TERT*-mut1-luc) (Figure 3B). These results suggested that rs2853669 inhibited E2F1 binding to the *TERT* promoter. Taken together, the data suggest that the rs2853669 variant induces *TERT* transcription levels by blocking E2F1 binding to its promoter.

### DNA methyltransferase 1 and histone deacetylase 1 are involved in E2F1-mediated down-regulation of TERT transcription levels in the absence of the rs2853669 variant

**Figure 4:**
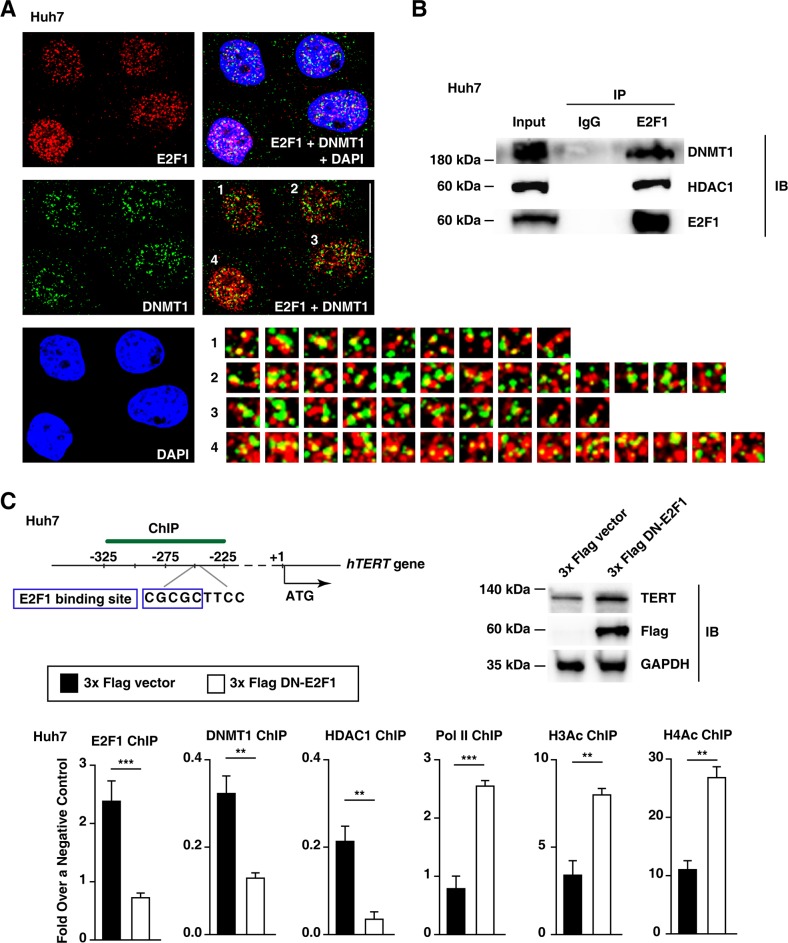
Epigenetic changes in the *TERT* promoter are involved in increased *TERT* transcription levels in Huh7 cells **A.** Immunofluorescence staining of E2F1 (red), DNMT1 (green), and DAPI (blue) in Huh7 cell lines. Scale bar, 20 μm. The number-labeled boxes indicate the areas of protein co-localization (yellow). DAPI, 4′,6-diamidino-2-phenylindole. **B.** Huh7 cell nuclear extracts or immunoprecipitated products (IP) generated using control IgG and E2F1 antibodies were subjected to immunoblot analysis (IB) using the antibodies indicated on the right. **C.** Immunoblot assay and ChIP experiments using the *TERT* promoter from each 3x Flag empty vector- or 3x Flag DN-E2F1-ectopic expressed Huh7 cell line. Pol II, RNA polymerase II; H3Ac and H4Ac, acetylated histones H3 and H4. The data are shown as the mean ± SEM, **P < 0.01, *** *P* < 0.005.

E2F1 represses gene transcription levels by recruiting DNA methyltransferase 1 (DNMT1) [27]. Because DNMT1 acetylation often induces DNMT1 degradation, histone deacetylase 1 (HDAC1) stabilizes DNMT1 by interacting with DNMT1 [27, 28]. Immunostaining analyses revealed that E2F1 can interact with DNMT1, and the co-localization of DNMT1 and E2F1 was clearly observed in Huh7 cell nuclei (Figure 4A). In addition, co-immunoprecipitation analyses further demonstrated that both DNMT1 and HDAC1 [27, 28] interact with E2F1 (Figure 4B). These data suggest that the DNMT1, HDAC1, and E2F1 work in concert to facilitate *TERT* transcription level repression.

Our ChIP data demonstrated that reducing endogenous E2F1 expression levels via a dominant-negative E2F1 (DN-E2F1) decreases both DNMT1 and HDAC1 binding to the *TERT* promoter in Huh7 cells (Figure 4C). On the contrary, the binding of the transcription activation-associated factors, RNA polymerase II (Pol II) and acetylated histones H3 and H4 (H3Ac and H4Ac), increased at the *TERT* promoter region 318–227 bp upstream of ATG after the dominant-negative-mediated inhibition of E2F1 (Figure 4C). Taken together, E2F1 binding to the *TERT* promoter enhanced DNMT1 and HDAC1 recruitment in Huh7 cells.

### Inhibiting E2F1 binding to the TERT promoter does not increase TERT transcription levels in HepG2 cells with the rs2853669 variant

**Figure 5:**
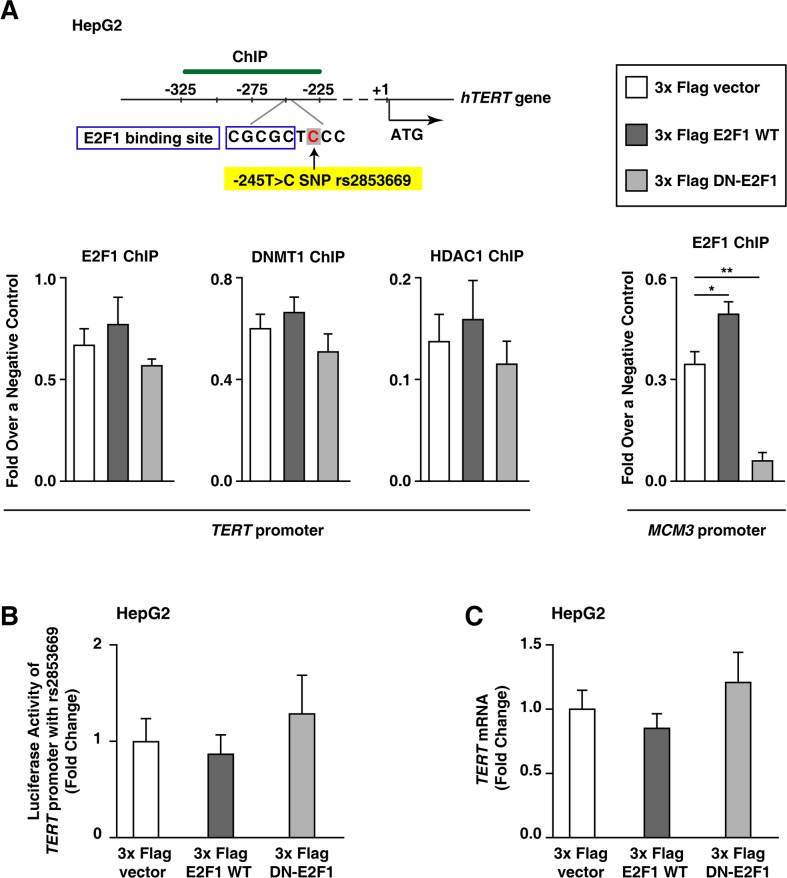
E2F1 does not repress *TERT* transcription in HepG2 cells with rs2853669 **A.** ChIP experiments using *TERT* promoter from each 3x Flag empty vector–, 3x Flag E2F1–, or 3x Flag DN-E2F1–ectopic expressed HepG2 cell line. Primers for *Minichromosome maintenance complex component 3* (*MCM3*) were used as an internal control. **B, C.** Luciferase assay (B) and qPCR (C) using 3x Flag empty vector–, 3x Flag E2F1–, or 3x Flag DN-E2F1–ectopic expressed HepG2 cells. The data are shown as the mean ± SEM, *P < 0.05, ** *P* < 0.01.

To examine whether the rs2853669 variant (−245T > C) affects E2F1-mediated DNMT1 and HDAC1-binding to the *TERT* promoter, we analyzed the interaction between E2F1 and the rs2853669 variant in HepG2 cells, which is a HCC cell line with the rs2853669 variant (Supplementary Figure 3). The *TERT* promoter sequence (from 367 bp upstream of ATG to 35 bp downstream of ATG [26]; Supplementary Figure 3) does not differ between HepG2 and Huh7 cells, except that the former includes the rs2853669 variant, and the latter does not. ChIP analyses after ectopic expression of WT E2F1 (increased E2F1) or DN-E2F1 (decreased E2F1) showed no difference in E2F1-binding to the *TERT* promoter compared with ectopic expression of an empty vector in HepG2 cells (Figure 5A). The difference in DNMT1 or HDAC1 binding to the *TERT* promoter was also negligible between E2F1-up-regulated HepG2 cells and E2F1-down-regulated HepG2 cells (Figure 5A). This result is consistent with the observation that the luciferase activity of the *TERT* promoter with the rs2853669 variant did not exhibit a statistically significant difference when E2F1 expression was up-regulated and when E2F1-binding to the *TERT* promoter was inhibited (Figure 5B). As expected, the *TERT* mRNA expression levels in HepG2 did not increase even though endogenous E2F1 activity was inhibited by DN-E2F1 mutant overexpression (Figure 5C), which indicates that rs2853669 is responsible for evading E2F1-induced down-regulation of *TERT* expression levels.

### The rs2853669 variant is associated with a methylated TERT promoter in HCC cell lines and HCC tumors

**Figure 6:**
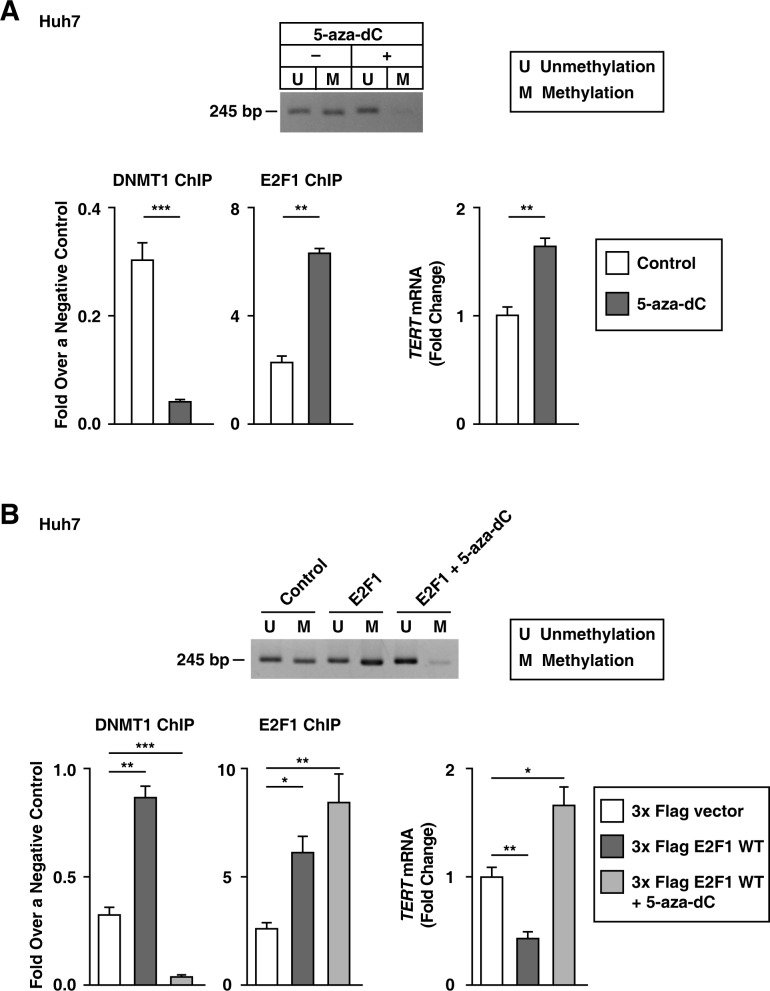
Blocking DNMT1 activity increases *TERT* transcription levels in Huh7 cells without rs2853669 **A, B.** MS-PCR using the primer for the *TERT* CpG island MSP targeting site, qPCR, and the ChIP experiment using 5-aza-dC-treated Huh7 cells (A), and a 3x Flag empty vector–, 3x Flag E2F1–, or 3x Flag E2F1 combined with 5-aza-dC-treated Huh7 cells (B) The data are shown as the mean ± SEM, *P < 0.05, ** *P* < 0.01, *** *P* < 0.005. MS-PCR, methylation specific PCR.

Next, we validated that the role of E2F1 as a *TERT* transcription repressor was dependent on DNMT1 activity. We found that 5-aza-2-deoxycytidine (5-aza-dC)-treated Huh7 cells had lower *TERT* promoter methylation levels and increased *TERT* transcription levels, despite an increase in the binding of E2F1 to the *TERT* promoter (Figure 6A). Since previous studies show that 5-aza-dC inhibits DNMT1 activity [29] and that *TERT* promoter methylation can inhibit the binding of TERT repressors [30], it is possible that DNMT1 regulates the action of E2F1 as a TERT transcription repressor in Huh7 cells. We further examined whether E2F1 localized DNMT1 to the *TERT* promoter containing the site at −245 bp relative to ATG. E2F1 overexpression increased DNMT1 binding to the *TERT* promoter and decreased the *TERT* transcription level; however, concurrent 5-aza-dC treatment and E2F1 overexpression decreased DNMT1-binding to the *TERT* promoter and increased *TERT* transcription levels (Figure 6B). The rs2853669 variant disrupted the E2F1 binding-site, which was demonstrated through luciferase reporter and ChIP assays (Figures 3 and 5). Collectively, the rs2853669 variant aids in stimulating *TERT* transcription levels by preventing E2F1-mediated DNMT1 localization to the *TERT* promoter.

**Figure 7:**
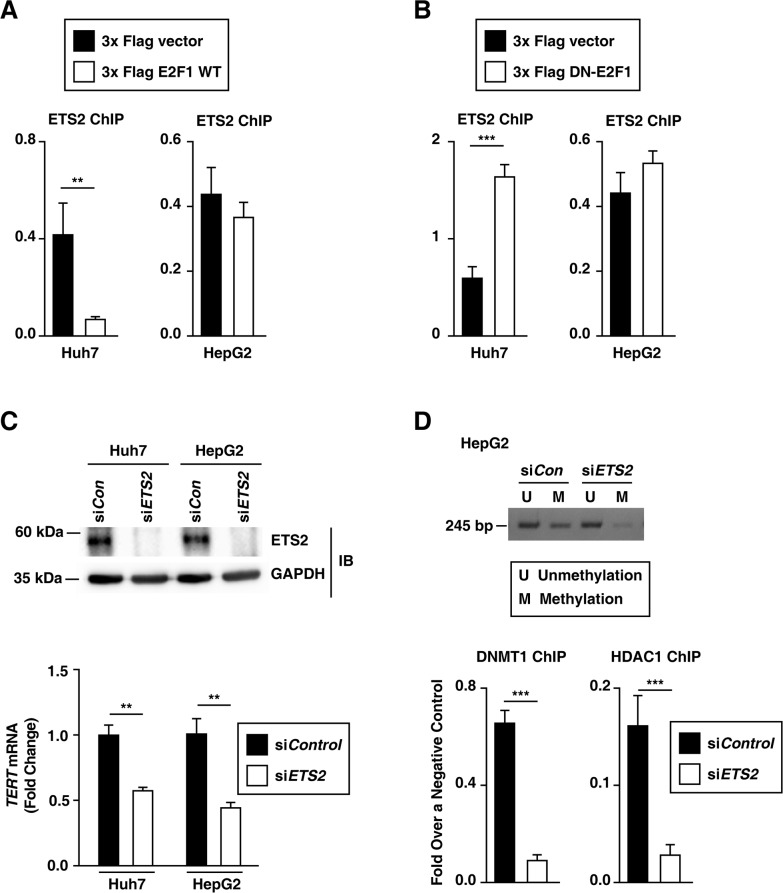
E2F1 and ETS2 regulate the rs2853669 variant-mediated *TERT* expression in HCC cells **A, B.** ETS2 ChIP experiments using the *TERT* promoter after ectopic expression of each 3x Flag empty vector and 3x Flag E2F1 (A) and a 3x Flag empty vector and 3x Flag DN-E2F1 (B) in Huh7 and HepG2 cells. **C.** Immunoblot assay and qPCR after siRNA-mediated *ETS2* knockdown in Huh7 and HepG2 cells. **D.** MS-PCR using the primer for the *TERT* CpG island MSP targeting site and ChIP experiment using siControl (siCon)- and siETS2-treated HepG2 cells. The data are shown as the mean ± SEM, **P < 0.01, *** *P* < 0.001. MS-PCR, methylation specific PCR.

The rs2853669 variant (the −245T > C) was not only located near the E2F1 binding site, but also overlapped with an ETS2 binding site at the *TERT* promoter [10] (Figure 1A). We first examined whether E2F1 affected ETS2 binding to the *TERT* promoter by ChIP assay (318–227 bp upstream of the ATG start site). The level of binding between ETS2 and the *TERT* promoter decreased in E2F1 overexpressed–Huh7 cells, whereas the level remained unchanged in E2F1-overexpressed HepG2 cells (Figure 7A). Moreover, in HepG2 cells, the level of ETS2 binding to the *TERT* promoter did not change when E2F1 binding to the *TERT* promoter was inhibited (Figure 7B). Interestingly, in Huh7 cells, deficient E2F1 activity increased ETS2 binding to the *TERT* promoter (Figure 7B). The rs2853669 variant may disrupt both E2F1 and ETS2 binding sites, and thus interfere with both E2F1 and ETS2 binding to the *TERT* promoter in HepG2 cells. Consistent with the previous studies [12], silencing *ETS2* by siRNA decreased *TERT* transcription levels in Huh7 and HepG2 cells (Figure 7C), which demonstrates that the ETS2 effects on *TERT* transcription activation differs from E2F1. Using a luciferase assay, we show that the rs2853669 variant increased *TERT* transcription activity in HCC cell lines (Figure 2A), which suggests that E2F1, not ETS2, dominantly affects the *TERT* promoter region containing the site at −245 bp relative to ATG. Altogether, E2F1 may occupy the *TERT* promoter region containing the variant site and then interfere with ETS2 binding to the *TERT* promoter.

Both HepG2 cells and Huh7 cells exhibited a −124C > T mutation [19], which creates an ETS2 binding site. Interestingly, *ETS2* knockdown decreased both DNMT1 and HDAC1 binding to the *TERT* promoter and further decreased *TERT* promoter methylation in HepG2 cells (Figure 7D). Blocking E2F1 alone failed to decrease DNMT1 binding to the *TERT* promoter in HepG2 cells (Figure 5A), which indicates that ETS2 is required for methylation of the *TERT* promoter region containing the rs2853669 variant (−245T > C). In Huh7 cells, blocking both E2F1 and ETS2 also decreased both DNMT1 and HDAC1 binding to the *TERT* promoter and decreased *TERT* promoter methylation Supplementary Figure 4). Thus, we conclude that both the rs2853669 variant (the co-target of E2F1 and ETS2) and −124C > T mutation (the ETS2 target) are involved in the modulation of methylation at the *TERT* promoter and further increased the *TERT* expression in HCC cells.

**Figure 8:**
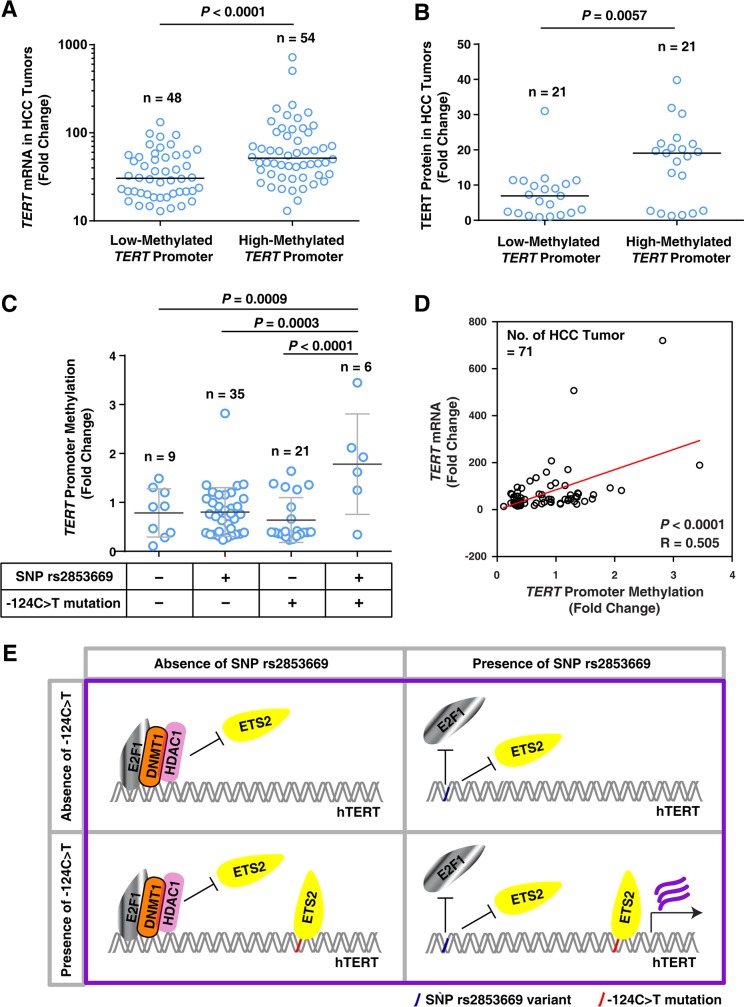
The rs2853669 variant combined with the −124C>T mutation is associated with *TERT* promoter methylation in HCC tumors **A.** Quantification of *TERT* mRNA levels in HCC tumors with a low-methylated- or high-methylated-*TERT* promoter. Horizontal bars indicate the median value. Samples were separated into 2 groups based on the median methylation level of *TERT* promoter. n = 102. **B.** Quantification of TERT protein expression in HCC tumors with a low-methylated- or high-methylated-*TERT* promoter. Horizontal bars indicate the median value. Samples were separated into 2 groups based on the median methylation level of *TERT* promoter. n = 42. **C.** Quantitation of *TERT* promoter methylation in HCC tumors without rs2853669 (−245T > C) and the −124C > T mutation or with the SNP rs285366 only, the −124C > T mutation only, or combination of both (−124C > T + SNP). **D.** Linear regression analysis of *TERT* promoter methylation levels (%) and *TERT* mRNA levels. R, Spearman's rank correlation coefficient. **E.** Molecular model illustrating the potential function of rs2853669 and −124C > T mutation in regulating *TERT* transcription.

We previously demonstrated that methylation in the *TERT* promoter region spanning from −270 bp to −31 bp upstream of the ATG start site correlates with high *TERT* expression levels and poor recurrence-free survival rates in HCC patients [31]. The present findings show that the rs2853669 variant (−245T > C) and −124C > T mutation combination increase *TERT* expression, which is involved in *TERT* promoter methylation as described in our previous report [31] (Figures 2 and 7). We propose that *TERT* expression is increased by *TERT* promoter methylation in HCC tumors with the rs2853669 variant and −124C > T mutation combination. The data show that the *TERT* promoter methylation is positively associated with *TERT* mRNA expression and TERT protein expression in HCC tumors (Figure 8A, 8B and Supplementary Figure 5), which is consistent with our previous data [31]. To examine whether the rs2853669 variant and −124C > T mutation combination is related to methylation of the *TERT* promoter containing the site at −245 bp relative to ATG, we quantified *TERT* promoter methylation levels in the four cases of HCC tumors; with rs2853669 variant alone, with the −124C > T mutation alone, with both the variant and mutation combination, and without neither the variation nor the mutation. We found that the *TERT* promoter methylation levels were greater in HCC tumors with the rs2853669 variant and −124C > T mutation combination compared with the HCC tumors without both the variation and mutation (*P* = 0.0009), the HCC tumors the with rs2853669 variant alone (*P* = 0.0003), and the HCC tumors with the −124C > T mutation alone (*P* < 0.0001) (Figure 8C). A positive correlation between the *TERT* promoter methylation level (270–31 bp upstream of the ATG start site) and the *TERT* mRNA expression level was also confirmed by linear regression analysis (*P* < 0.0001; Figure 8D). We showed that a combination of the rs2853669 variant (−245T > C) and −124C > T mutation were associated with poor survival rate in HCC patients (Figure 1). Taken together, these results suggest that this combination contributes to the poor survival rate of HCC through *TERT* promoter methylation-mediated *TERT* transcriptional activation (Figure 8D).

## DISCUSSION

Methylation at the *TERT* promoter has been observed in various tumor tissues and transformed cell lines [32]. Furthermore, it exhibits a positive association with high *TERT* expression levels and poor survival rates in patients with childhood brain tumors [30]. Our previous data show that *TERT* promoter methylation contributes to HCC progression by increasing TERT expression [31]. Here, our study demonstrates that *TERT* promoter methylation was induced by decreasing E2F1 binding (in the presence of rs2853669 (−245T > C)) combined with increasing ETS2 binding at the *TERT* promoter (in the presence of the −124C > T mutation). Notably, we found that the rs2853669 variant and −124C > T mutation combination is markedly associated with high *TERT* expression levels, poor overall survival rates, and poor recurrence-free survival rates in HCC patients. HCC patients with the combination also show greater *TERT* promoter methylation levels compared with HCC patients with the variant alone or mutation alone and HCC patients without both the variant and mutation, indicating that the variant combined with the mutation contributes to *TERT* promoter methylation, which leads to increased TERT expression, HCC mortality, and HCC recurrence.

The level of *TERT* expression is increased by the inhibition of E2F1 as a transcriptional repressor through the rs2853669 variant (−245T > C); however, the concurrent stimulation of ETS2 as a transcriptional activator through the −124C > T mutation is also required for increased *TERT* expression (Figure 8E). The ETS2 binding to the site adjacent to the variant site have been reported to induce c-MYC binding to the *TERT* promoter, thereby leading to an increase in *TERT* expression [12]. Previous reports suggested that rs2853669 disrupts the ETS2 binding site, thus reducing the c-MYC-induced *TERT* expression regulation [17, 33]. However, a recent finding showed that c-MYC knockdown can instead activate *TERT* promoter activity through enhanced binding of multiple transcription activators to the *TERT* promoter [34]. As the binding of a transcription factor to its promoter can lead to local structural modification, causing the removal of a preexisting component or recruitment of a new component, the binding of ETS2 to the *TERT* promoter exhibits the modulation of local structure in the promoter region [12]. A recent study by Bell RJ et al. also suggests that *TERT* promoter mutations (−124C > T and −146C > T) cooperate with native ETS sites to form high-order structures such as G-quadruplexes; as a result, these structures contribute to the recruitment of the multimeric GA-binding protein (GABP) transcription factor and to the up-regulation of TERT expression [35]. The −245T region is located on a ETS2 binding site, but this ETS2 binding site does not belong to the native ETS sites reported by Bell RJ. et al. [35]. This suggests that TERT expression is regulated by the cooperation of TERT transcription factor binding sites, including TERT promoter mutations, native ETS sites, and SNPs. Thus, it is possible that the rs2853669 and −124C > T mutation combination alters the structural modification of the *TERT* promoter to improve its activity.

Several SNPs in the telomere maintenance genes are highly associated with the survival rates in HCC patients [6]. According to the data regarding the association between HCC risk and SNPs in the *TERT* gene, the SNP rs13167280 (IVS3–24 C > T), located on the third intron of the *TERT* gene, is associated with a decreased risk of HCC progression, whereas no significant association is reported between rs2853669 (−245T > C) and HCC risk [6]. However, our current data demonstrate that the rs2853669 variant combined with the −124C > T mutation at the *TERT* promoter increases *TERT* expression, telomere length, and HCC mortality and recurrence rates. Thus, in order to better predict the prognosis of HCC patients, we suggest that studies of SNPs expand to incorporate the relationships and the clinical implications of SNPs when coupled with other SNPs or genetic alterations.

We show that rs2853669 (−245T > C) does not affect the luciferase promoter activity of the *TERT* reporter vector with the −146C > T mutation (Figure 2A) which is a rare mutation in our HCC patient cohorts (0 case out of 93 cases for the SMH cohort, and 1 case out of 72 cases for the KU cohort; Supplementary Tables 2 and 3). Therefore, the rs2853669 and −124C > T combination likely increases telomerase activity by elevating *TERT* expression levels, thereby elongating telomere length in HCC. In another study, this combination decreased *TERT* promoter activity, as assessed by luciferase activity, in urothelial carcinoma cell lines (T24 cell line and CLS-439 cell line) [17], thereby showing that rs2853669 may modulate the *TERT* promoter activity in a cell type-specific manner. Different cancer types have various TERT promoter mutation frequencies [10, 11, 17, 19, 20]. While 81.8% of urothelial carcinomas carry a −124C > T mutation and 17.8% a −146C > T mutation [17], HCC showed a −124C > T mutation in 93–100% of the cases and a −146C > T mutation in 6–10% of the cases in our study (Supplementary Tables 2 and 3) as well as in other reports [19, 20]. The interaction of *TERT* promoter mutations and ETS binding sites [35] can regulate the expression of TERT. On the same line, the binding sites for TERT transcription factors can cooperate with SNPs, leading to the activation of TERT via the recruitment of multiple TERT transcription factors. This is probably the reason for cancer-specific effects of SNPs on TERT expression. The rs2853669 variant and −124C > T mutation combination increased *TERT* promoter activity in four HCC cell lines, which indicates that rs2853669 is involved in regulating *TERT* promoter activity in cell types that are originated from HCC.

*TERT* promoter mutations are major genomic alterations in the step-by-step hepatocarcinogenic process, which is involved in HCC developed from chronic liver disease as well as from hepatocellular adenomas (HCA) [36, 37]. A recent report also showed that *TERT* promoter mutations are key determinants for HCC, as they are observed in low- or high- grade dysplastic nodules (LGDNs, HGDNs), while other mutations in ten cancer genes (CTNNB1, TP53, ARID1A, ARID2, NFE2L2, AXIN1, PIK3CA, KEAP1, RPS6KA3, and CDKN2A) have not been observed in cirrhotic livers, LGDNs, and or HGDNs [38]. Although a number of studies indicate that *TERT* promoter mutations may be a determinant for HCC, HCC tumors displaying increased TERT expression independent of the *TERT* mutation status also exist [37]. We showed that the rs2853669 polymorphism combined with *TERT* promoter mutations increased TERT expression compared to *TERT* promoter mutations only (Figure 2). Thus, it is possible that the SNP rs2853669 polymorphism combined with TERT promoter mutations, as well as alternative causes, are part of the mechanism responsible for the increase in TERT expression.

In conclusion, we report for the first time that a common variant of the *TERT* gene, rs2853669, is significantly associated with a high risk of death and cancer recurrence in patients with liver cancer, and that the rs2853669 variant (−245T > C), combined with the −124C > T mutation, mediates *TERT* transcriptional activity by modulating the binding of both E2F1 and ETS2, which is responsible for the high risk of HCC. Our study suggests that the rs2853669 variant combined with the −124C > T mutation in the *TERT* promoter is a novel risk factor for poor prognosis in liver cancer.

## MATERIALS AND METHODS

### Human samples

The Seoul National University Institutional Review Board (SNUIRB No. E1308/001-035) approved this study (Supplementary Materials).

### DNA sequencing

Genomic DNA was extracted from HCC tumors and corresponding non-tumorous tissues. The genomic DNA samples from paraffin-embedded tissues and frozen tissues were isolated using the Arcturus PicoPure DNA Extraction Kit (Applied Biosciences, Foster City, CA, USA) and NucleoSpin^®^ TriPrep Kit (Macherey-Nagel, Düren, Germany; 740966.250), respectively, in accordance with the instructions of each manufacturer (Supplementary Materials).

### Quantification of telomere fluorescence levels using immunoFISH

An immunoFISH protocol [39, 40] was used with modifications (Supplementary Materials).

### Quantification of TERT mRNA expression levels by quantitative real-time PCR

The total RNA was isolated using the NucleoSpin^®^ TriPrep Kit (Macherey-Nagel, Düren, Germany; 740966.250), and cDNA was synthesized using TOPscript™ RT Drymix (dT18) (Enzynomics, Daejeon, Korea; RT200) (Supplementary Materials).

### Cell culture and treatment

The HCC cell lines (Huh7, Hep3B, HepG2, and SNU-449) were obtained from the Korean Cell Line Bank (KCLB, Seoul, Korea) (Supplementary Materials).

### Luciferase reporter assay

For the luciferase reporter assay, 1.5 × 10^5^ HCC cells were seeded onto 6-well plates and transfected using Fugene^®^ 6 (Roche, Basel, Switzerland) and 1 μg of the wild-type *TERT* promoter-luciferase construct with or without the variant rs2853669 (−245T > C) or *TERT* promoter mutation (−124C > T or −146C > T). The wild-type (WT) *TERT* promoter was subcloned into the pGL3 luciferase empty vector (Promega, Madison, WI, USA) (Supplementary Materials).

### Immunofluorescence assay

The cells were fixed using 4% paraformaldehyde, permeabilized using 0.5% Triton X-100 in PBS, blocked with 20% NGS (Normal Goat Serum) and treated with a mouse monoclonal anti-DNMT1 antibody (1:500, Abcam, Cambridge, MA, USA; ab13537) as well as a rabbit polyclonal anti-E2F1 antibody (1:500, ab6302; Cell Signaling) overnight at 4°C. After washing and incubating in a secondary antibody for 1 hour, the slides were mounted using a medium containing DAPI (Vector Laboratories). The images were collected using a confocal microscope (LSM 700; Carl Zeiss, Oberkochen, Germany). The image analyses were performed using Image-Pro plus 6.0 software (Media Cybernetics, Inc., Rockville, MD, USA).

### Immunoblot analysis

A total of 2 × 10^5^ cells were boiled for 5 minutes in 2× SDS sample buffer (100 mM Tris–HCl [pH 6.8], 4% SDS, 0.2% bromophenol blue, 20% glycerol, and 200 mM β-mercaptoethanol) and subjected to SDS–PAGE as well as western blotting (Supplementary Materials).

### Chromatin immunoprecipitation

Chromatin immunoprecipitation (ChIP) experiments were performed as previously described [29] with certain modifications (Supplementary Materials).

### TERT promoter methylation assay

Two micrograms of genomic DNA was treated with sodium bisulfite, and the bisulfite-converted DNA was purified using an EpiTect Bisulfite Kit (Qiagen) in accordance with the manufacturer's instructions (Supplementary Materials).

### Statistical analysis

The data were analyzed using R software (www.r-project.org) and GraphPad Software version 4.0 (GraphPad Software Inc., San Diego, CA, USA). The survival data were estimated using the Kaplan–Meier method, and the differences in the survival rates were compared using the log-rank test. The mRNA expression levels and telomere lengths were analyzed using the Mann–Whitney rank sum test, and the promoter activity and ChIP assay were analyzed using a two-tailed *t* test. The experiments were independently repeated at least three times. The significance values were *P < 0.05, **P < 0.01, and ***P < 0.001.

## SUPPLEMENTARY MATERIALS AND METHODS FIGURES AND TABLES


