# Supplementary material for: The TERT promoter SNP rs2853669 decreases E2F1 transcription factor binding and increases mortality and recurrence risks in liver cancer
Source: Oncotarget. 2015 Nov 9;7(1):684–99. doi: 10.18632/oncotarget.6331 (PMC4808026; doi:10.18632/oncotarget.6331)
Supplement: Supplementary file 1 [file oncotarget-07-0684-s001.pdf]

SUPPLEMENTARY MATERIALS AND METHODS

Human samples

For measurement of telomere fluorescence levels, 93 surgically resected paraffin-embedded HCC tumor tissue samples and corresponding non-tumorous liver tissue samples were analyzed. For measurement of the *TERT* mRNA expression level, 72 surgically resected frozen HCC tumor tissue samples and corresponding non-tumorous liver tissue samples were analyzed. For methylation-specific PCR analysis, we used 102 samples of HCC tumors, including 24 samples of HCC tumors used in a previous report [1] and 78 samples of HCC tumors used in previous report [2]. The cases were prospectively and consecutively identified at Seoul St. Mary's Hospital of the Catholic University of Korea between 2005 and 2009 (93 paraffin-blocked tissue samples) and between 2011 and 2013 (19 frozen tissue samples), as well as at Korea University Guro Hospital (KU Guro Gene Bank 2013-020) between 2010 and 2013 (53 frozen tissue samples). We designated 93 paraffin-blocked tissues from Seoul St. Mary's Hospital of the Catholic University as the SMH cohort, and a total of 19 frozen tissues from Seoul St. Mary's Hospital of the Catholic University and 53 frozen tissues from Korea University Guro Hospital as the KU cohort, respectively. The histological grade of tumor differentiation was defined according to the Edmondson–Steiner grading system [3].

DNA sequencing

The *TERT* promoter containing rs2853669 (-245T) [4], -124C [5], and -146C [5] (chr5: 1,295,349; chr5: 1,295,228; chr5: 1,295,250, respectively; hg19 assembly) was amplified using hTaq polymerase (Solgent, Daejeon, Korea) with 10 ng of genomic DNA containing each primer (forward primer: GGCCGATTCGACCTCTCT; reverse primer: AGCACCTCGCGGTAGTGG) [6]. Tissues carrying the rs2853669 variant displayed CC or CT genotypes (Supplementary Tables 2 and 3) [5]. The -124C > T and -146C > T mutations are somatic mutations at positions -124 bp and -146 bp from ATG start site of the *TERT* gene, respectively [5]. After purification of a 489 bp PCR-amplified product with the NucleoSpin DNA Clean-up Kit (Macherey-Nagel, Düren, Germany; 740609.250), the *TERT* promoter region was analyzed by Sanger sequencing (Macrogen, Seoul, Korea). Common genetic variants and all mutations were visually confirmed by Chromas Lite 2.1 software (Technelysium, South Brisbane, Australia) from both strands of directly sequenced PCR products.

Quantification of telomere fluorescence level with immunoFISH

Paraffin-embedded sections were deparaffinized with xylene and rehydrated using an ethanol gradient. Antigen retrieval was performed by boiling sections in 100 mM sodium citrate (pH 6.0) for 10 minutes in a microwave oven. After permeabilization with proteinase K (15 µg/mL in phosphate-buffered saline [PBS; pH 7.4]) at 37°C for 20 minutes and 50% formamide treatment, the samples were fixed using 4% formaldehyde followed by air drying. The samples were then denatured for 6 minutes at 90°C followed by hybridization for 2 hours at room temperature in the dark. The hybridization solution contained 70% formamide in 2× saline-sodium citrate buffer, 5% $MgCl_2$, 0.25% blocking reagent (Roche, Basel, Switzerland), 15.4 nM of Cy3-labeled telomere peptic nucleic acid (PNA) probe (TelC–Cy3: CCCTAACCCTAACCCTAA; Panagene, Korea), and 18.4 nM of fluorescein amidite (FAM)-labeled centromere PNA probe (Cent–FAM: AAACTAGACAGAAGCATT; Panagene). After the slide had been washed, it was rinsed with PBS containing 4′,6-diamidino-2-phenylindole (DAPI) and then mounted with DAPI mounting medium (Vector Laboratories, Burlingame, CA, USA). A centromeric probe was used as an internal control. The telomere fluorescence level refers to the ratio of telomere fluorescence intensity to centromere fluorescence intensity. The same exposure time was used for the quantification of telomere fluorescence level. The telomere fluorescence level was calculated by dividing the telomere fluorescence level of the tumor tissue by that of the corresponding non-tumorous tissue at five random fields. Image analysis was performed using Image-Pro plus 6.0 software (Media Cybernetics, Inc., Rockville, MD, USA).

Quantification of TERT mRNA expression levels by quantitative real-time PCR

PCRs were performed using the TOPreal™ qPCR 2× PreMIX (SYBR Green with high ROX) (Enzynomics, Daejeon, Korea; RT501M) and an ABI Prism 7300 thermal cycler (Applied Biosystems, Foster City, CA, USA). Gene expression was normalized to that of *β-actin*. The primer sequences for each gene were as follows: *TERT* (forward primer: GCCTTCAAGAGCCACGTC; reverse primer: CCACGAACTGTCGCATGT) [7]; and *β-actin* (forward primer: GCAAAGACCTGTACGCCAACA; reverse primer: TGCATCCTGTCGGCAATG). A dissociation step was added to check for primer specificity.

Cell culture and treatment

All four cell lines were tested for mycoplasma contamination. The HCC cell lines were grown in Dulbecco's modified Eagle's medium (Welgene, Gyeongsan-si, Korea) supplemented with 10% fetal bovine serum (GenDepot, Barker, TX, USA) in a humidified 5% CO_2 incubator at 37°C [1]. For DNA methyltransferase (DNMT1) activity inhibition, cells were treated with 10 µM of 5-aza-dC (Sigma–Aldrich, St. Louis, MO, USA) [1]. Wild-type E2F1 and dominant-negative E2F1 (DN-E2F1) were subcloned into 3x Flag pCMV-10 (Sigma–Aldrich, St. Louis, MO, USA). Replacing two amino-acid residues in the DNA binding domain of E2F1 (introducing glutamic acid and phenylalanine residues at positions 132–133: LN132–133EF, here designated DN-E2F1) creates a dominant-negative construct that suppresses endogeneous E2F1 activity [8]. The neomycin-resistant clones isolated through G418 selection (1 mg/mL) (Sigma–Aldrich, USA) were used in the experiments. For transient gene depletion, nontargeting control siRNA (sc-37007) and *ETS2* siRNA (sc-37855) were purchased from Santa Cruz Biotechnologies (Santa Cruz Biotechnology, Dallas, TX, USA) and transfected into cells with Lipofectamine RNAiMax (Life Technologies, Foster City, CA, USA) at a 25 nM concentration [7]. The immunoblot assay, Q-RT-PCR, the ChIP assay, and MS-PCR were performed 4 days after siRNA transfection, 5-aza-dC treatment, or ectopic plasmid expression.

Luciferase reporter assay

The pGL3-wild-type *TERT* promoter plasmid was used as the template to generate mutant constructs through site-directed mutagenesis. The original and converted primer sequences are as follows: *TERT* promoter with the -124C > T mutation, CCCCGGCCCAGCCCCCTCCGGGCCCTCCCAG to CCCCGGCCCAGCCCC**T**TCCGGGCCCTCCCAG (109–139 bp upstream of the ATG start site); *TERT* promoter with the -146C > T mutation, CCCGTCCCGACCCCTCCCGGGTCCCCGGCCC to CCCGTCCCGACCCCT**T**CCGGGTCCCCGGCCC (131–161 bp upstream of ATG start site); E2F1 for *TERT*-luc mut1, CGCCCAGGACCGCGCTTCCCACGTGGCG to CGCCCAGGACCGC**CT**TTCCCAC GTGGCG (234–261 bp upstream of ATG start site); E2F1 for *TERT*-luc mut2, CCGCCTCCTCCGCGCGGACCCCGCCCCG to CCGCCTCCTCCGC**CT**GGACCCCGCCCCG (158–185 bp upstream of the ATG start site); E2F1 for *TERT*-luc mut4 (*TERT* promoter with the rs2853669 variant), GCCCAGGACCGCGCTTCCCACGTGGCGGAGG to GCCCAGGACCGCGCT**C**CCCACG TGGCGGAGG (230–260 bp upstream of the ATG start site). The bold text indicates the converted sequence. *TERT*-luc mut3 includes both mutations in *TERT*-luc mut1 and *TERT*-luc mut2. A total of 20 ng of *Renilla* luciferase (Promega, Madison, WI, USA) was co-transfected with each sample as an internal control for transfection efficiency. After 48 hours, the luciferase activity was quantified using a Dual-Luciferase® Reporter assay system (Promega, USA). All experiments were repeated three times.

Immunoblot analysis

Glyceraldehyde-3-phosphate dehydrogenase (GAPDH) was used as a loading control. TERT-specific (1:300, Rockland Immunochemicals, Gilbertsville, PA, USA; 600–401-252), E2F1-specific (1:1000, Cell Signaling Technology, Danvers, MA, USA; #3742), ETS2-specific (1:1000, Santa Cruz Biotechnology, Dallas, TX, USA; sc-351), Flag-specific (1:3000, Sigma–Aldrich, St.Louis, MO, USA; F 3165), or GAPDH-specific (1:2000, Santa Cruz Biotechnology; sc-47724) antibodies were diluted in 5% skim milk with TBS-Tween 20 and incubated overnight at 4°C, followed by washing and incubation in HRP-conjugated secondary antibodies (1:1000, Abcam, Cambridge, MA, USA; ab131368 or ab131366). Chemiluminescence images were acquired using a FUSION-SOLO imager (Vilber Lourmat, Marne La Vallée, France).

Chromatin immunoprecipitation

The cells were fixed using 1% paraformaldehyde while slowly shaking for 20 minutes at room temperature. Then, the cells were rinsed twice with ice-cold PBS; resuspended in 400 µl micrococcal nuclease buffer (50 mM Tris–HCl [pH8.0], 5 mM CaCl, 100 µg/ml BSA, 10 mM KCl, and protease inhibitor cocktail (Roche, Basel, Switzerland; 4693159001)); disrupted with 2-mm-diameter zirconium beads (Watson); and subjected to micrococcal nuclease digestion (NEB, M0247S). Chromatin (150 µg) was collected and diluted in dilution buffer (0.1% NP-40, 2 mM EDTA, 150 mM NaCl, 20 mM Tris–HCl [pH 8.0], protease inhibitor cocktail [Roche], and phosphatase inhibitor cocktail (Calbiochem, Darmstadt, Germany)) followed by pre-clearing with 2 µg sheared salmon sperm DNA, 10 µL preimmune serum (sc-2027, Santa Cruz), and Dynabeads Protein G (Life Technologies, Foster City, CA, USA; 1004D) for 2 hours at 4°C. After immunoprecipitation, 20 µL Dynabeads Protein G was added, and the incubation was continued for 2 hours. The Dynabeads were washed sequentially for 10 minutes each in TSE 1 (0.1% SDS, 1% Triton X-100, 2 mM EDTA, 20 mM Tris–HCl, and 150 mM NaCl), TSE II (0.1% SDS, 1% Triton X-100, 2 mM EDTA, 20 mM Tris–HCl, and 500 mM NaCl), and buffer III (0.25 M LiCl, 1% NP-40, 1% deoxycholate, 1 mM EDTA, and 10 mM Tris–HCl). The beads were then washed thrice with TE buffer and

extracted using 1% SDS and 0.1 M $NaHCO_3$. The eluates were heated at 65°C overnight to reverse the formaldehyde cross-linking. The DNA fragments were purified using the NucleoSpin DNA clean-up kit (Macherey-Nagel, Düren, Germany; 740609.250). The PCR amplification products were quantified by qPCR (ABI 7300; Applied Biosystems, Foster City, CA, USA) using primers specific to the *TERT* promoter regions. The ChIP primer sequences are as follows: a primer set for the human *TERT* promoter (forward primer: CGGAGCAGCTGCGCTGTC; reverse primer: GTCCCTCCGCCACGTGGGAAG), a primer set for human *TERT* intron (forward primer: TGAGGGCTGAGAAGGAGTGT; reverse primer: CACGATAGACGACGACCTCA), and a primer set for human *MCM3* promoter (forward primer: TCTTTGGCAGCGGGCAT; reverse primer: CGCAGCTCCACATCGTCC). PCR amplification was normalized to the *TERT* intron. ChIP for E2F1 were performed using *MCM3* promoter as a positive control [8]. The antibodies used were as follows: rabbit polyclonal anti-E2F1 (1:150, Cell Signaling Technology, Danvers, MA, USA; #3742), mouse monoclonal anti-DNMT1 (1:150, Abcam, Cambridge, MA, USA; ab13537), rabbit polyclonal anti-HDAC1 (1:50, Santa Cruz Biotechnology, Dallas, TX, USA; sc-7872), mouse monoclonal anti-RNA polymerase II (1:100, Covance, Princeton, NJ, USA; MMS-126R), rabbit polyclonal anti-ETS2 (1:100, Santa Cruz Biotechnology, Dallas, TX, USA; sc-351), rabbit polyclonal anti-H3Ac (1:100, Upstate Bio- technology, Lake Placid, NY, USA; 06–599), and rabbit polyclonal anti-H4Ac (1:100, Upstate Bio- technology; 06–866).

TERT promoter methylation assay

The purified DNA was amplified using methylation-specific primers. The methylation-specific primers were as follows: primers for unmethylated *TERT* DNA (forward primer: GTGGGTATAGATGTTTAGGATTGT; reverse primer: CCACATACACAACAAAACACAACA) and primers for methylated *TERT* DNA (forward primer: GGGTATAGACGTTTAGGATCGC; reverse primer: CGTACGCAACAAAACGCAACG). [9] The following PCR conditions were used: 95°C for 15 minutes and 95°C for 30 seconds; next, 40 cycles of 95°C for 30 seconds, 60°C for 40 seconds, and 72°C for 30 seconds; and finally, 10 minutes at 72°C. The PCR mixture contained l × hTaq buffer (Solgent, Daejeon, Korea) with 1 × Band Doctor, 20 pmol of each primer, 0.2 mM dNTPs, and 2 units of hTaq polymerase (Solgent). The reactions were analyzed using ethidium bromide-stained 2.5% agarose gels in 1 × Tris-borate EDTA buffer. The MSP primers were reported previously. [9] The MSP products were subcloned into the pGEM-T easy vector (Promega, USA) in accordance with the manufacturer's instructions. The plasmid DNAs were purified using a plasmid DNA purification kit (Intron Biotechnology, Sungnam, Korea) followed by sequencing (Macrogen, Seoul, Korea).

The *TERT* promoter methylation levels were determined by methylation specific quantitative PCR (MSqPCR) as previously described [10–12] with certain modifications. Methylation levels of the *TERT* promoter (the region is from 270 bp upstream of the ATG start site to 31 bp upstream of the ATG start site) were calculated as the fraction of methylated molecules in the total methylated and unmethylated DNA, which was described in a previous report [10, 11]. The myogenic differentiation 1 (MYOD1) gene was the internal reference gene [13]; The *MYOD1* gene primers (forward primer: CCAACTCCAAATCCCCTCTCTAT; reverse primer: TGATTAATTTAGATTGGGTTTAGAGAAGGA) were located in a region without the CpG nucleotide; thus, the cytosine was converted into uracil using a bisulfite treatment, and the primers for *MYOD1* bound only to the bisulfite-modified DNA.

SUPPLEMENTARY REFERENCES

1. Lim SO, Gu JM, Kim MS, Kim HS, Park YN, Park CK, Cho JW, Park YM and Jung G. Epigenetic changes induced by reactive oxygen species in hepatocellular carcinoma: methylation of the E-cadherin promoter. Gastroenterology. 2008; 135:2128-2140, 2140 e2121-2128.
2. Ko E, Jung ES and Jung G. Telomerase reverse transcriptase promoter methylation is related to a risk of recurrence in hepatocellular carcinoma. Hepatology. 2015.
3. Edmondson HA and Steiner PE. Primary carcinoma of the liver: a study of 100 cases among 48,900 necropsies. Cancer. 1954; 7:462-503.
4. Bojesen SE, Pooley KA, Johnatty SE, Beesley J, Michailidou K, Tyrer JP, Edwards SL, Pickett HA, Shen HC, Smart CE, Hillman KM, Mai PL, Lawrenson K, Stutz MD, Lu Y, Karevan R, et al. Multiple independent variants at the TERT locus are associated with telomere length and risks of breast and ovarian cancer. Nat Genet. 2013; 45:371-384, 384e371-372.
5. Rachakonda PS, Hosen I, de Verdier PJ, Fallah M, Heidenreich B, Ryk C, Wiklund NP, Steineck G, Schadendorf D, Hemminki K and Kumar R. TERT promoter mutations in bladder cancer affect patient survival and disease recurrence through modification by a common polymorphism. Proc Natl Acad Sci U S A. 2013; 110:17426-17431.
6. Killela PJ, Reitman ZJ, Jiao Y, Bettegowda C, Agrawal N, Diaz LA, Jr., Friedman AH, Friedman H, Gallia GL, Giovanella BC, Grollman AP, He TC, He Y, Hruban RH, Jallo GI, Mandahl N, et al. TERT promoter mutations occur

frequently in gliomas and a subset of tumors derived from cells with low rates of self-renewal. Proc Natl Acad Sci U S A. 2013; 110:6021-6026.

7. Hoffmeyer K, Raggioli A, Rudloff S, Anton R, Hierholzer A, Del Valle I, Hein K, Vogt R and Kemler R. Wnt/beta-catenin signaling regulates telomerase in stem cells and cancer cells. Science. 2012; 336:1549-1554.

8. Taubert S, Gorrini C, Frank SR, Parisi T, Fuchs M, Chan HM, Livingston DM and Amati B. E2F-dependent histone acetylation and recruitment of the Tip60 acetyltransferase complex to chromatin in late G1. Mol Cell Biol. 2004; 24:4546-4556.

9. Dessain SK, Yu H, Reddel RR, Beijersbergen RL and Weinberg RA. Methylation of the human telomerase gene CpG island. Cancer Res. 2000; 60:537-541.

10. Kitange GJ, Carlson BL, Mladek AC, Decker PA, Schroeder MA, Wu W, Grogan PT, Giannini C, Ballman KV, Buckner JC, James CD and Sarkaria JN. Evaluation of MGMT promoter methylation status and correlation with temozolomide response in orthotopic glioblastoma xenograft model. J Neurooncol. 2009; 92:23-31.

11. Banelli B, Brigati C, Di Vinci A, Casciano I, Forlani A, Borzi L, Allemanni G and Romani M. A pyrosequencing assay for the quantitative methylation analysis of the PCDHB gene cluster, the major factor in neuroblastoma methylator phenotype. Lab Invest. 2012; 92:458-465.

12. Usadel H, Brabender J, Danenberg KD, Jeronimo C, Harden S, Engles J, Danenberg PV, Yang S and Sidransky D. Quantitative adenomatous polyposis coli promoter methylation analysis in tumor tissue, serum, and plasma DNA of patients with lung cancer. Cancer Res. 2002; 62:371-375.

13. Zhang W, Bauer M, Croner RS, Pelz JO, Lodygin D, Hermeking H, Sturzl M, Hohenberger W and Matzel KE. DNA stool test for colorectal cancer: hypermethylation of the secreted frizzled-related protein-1 gene. Dis Colon Rectum. 2007; 50:1618-1626; discussion 1626-1617.

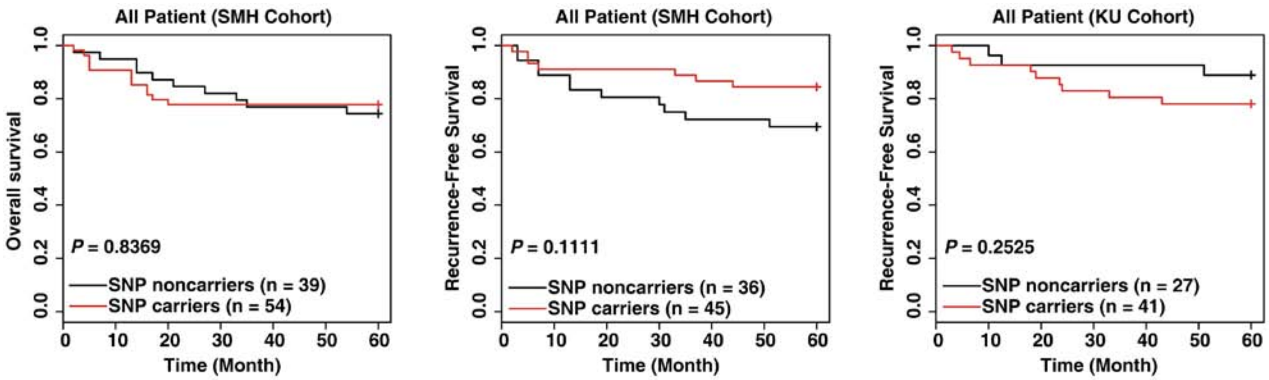

Supplementary Figure S1: Survival analysis for hepatocellular carcinoma patients carrying the rs2853669 variant. Kaplan–Meier analysis of differences in overall and recurrence-free survival for rs2853669 carriers and non-carriers. The patients were from the SMH cohort (left and middle) and the KU cohort (right).

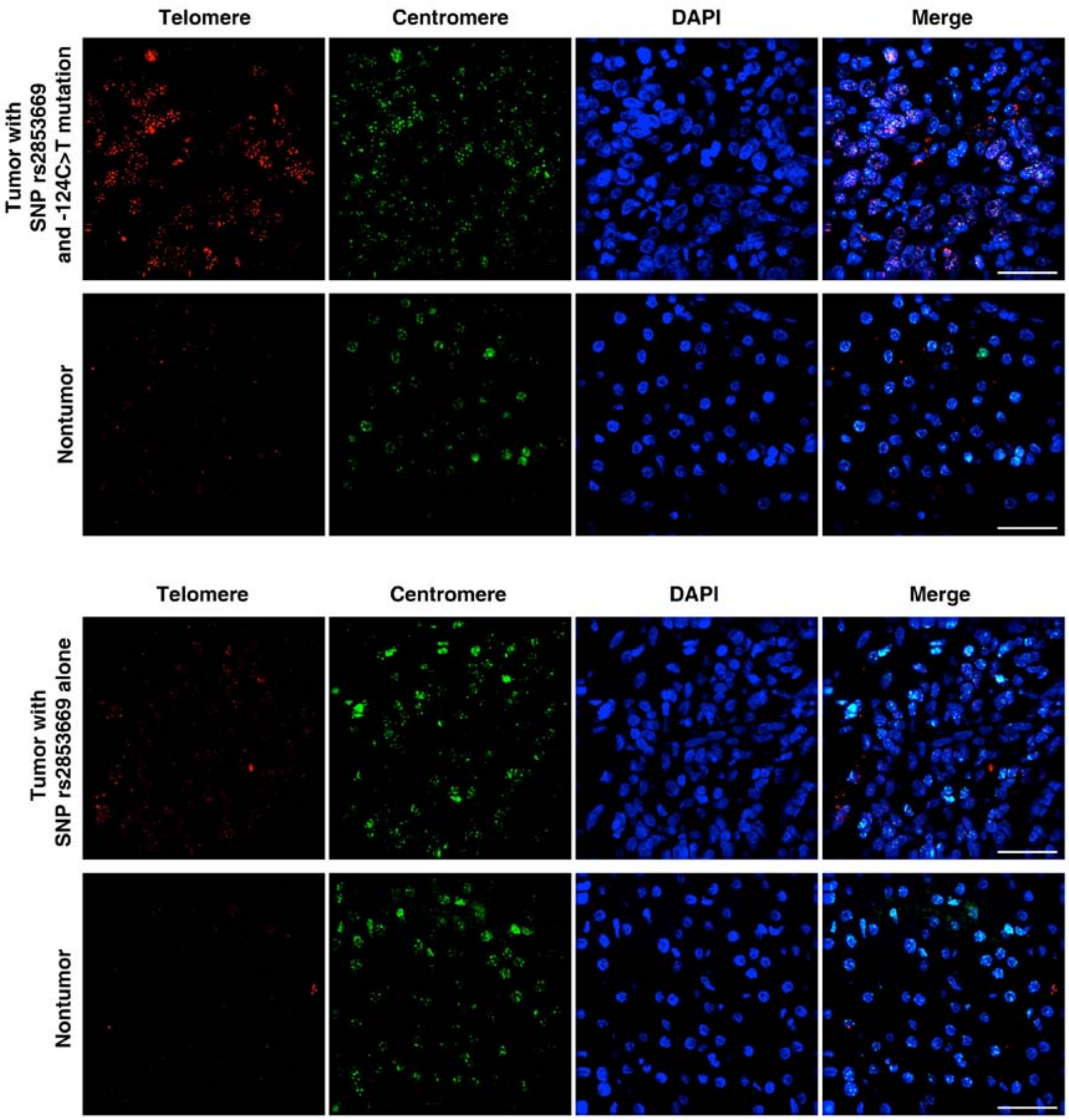

Supplementary Figure S2: ImmunoFISH image of telomere (red), centromere (green), and DAPI (blue) in HCC tumor tissue (SNP carriers with mutation, top; SNP carriers without mutation, bottom) and corresponding nontumor tissue. Scale bars, 40 µm. DAPI, 4′,6-diamidino-2-phenylindole.

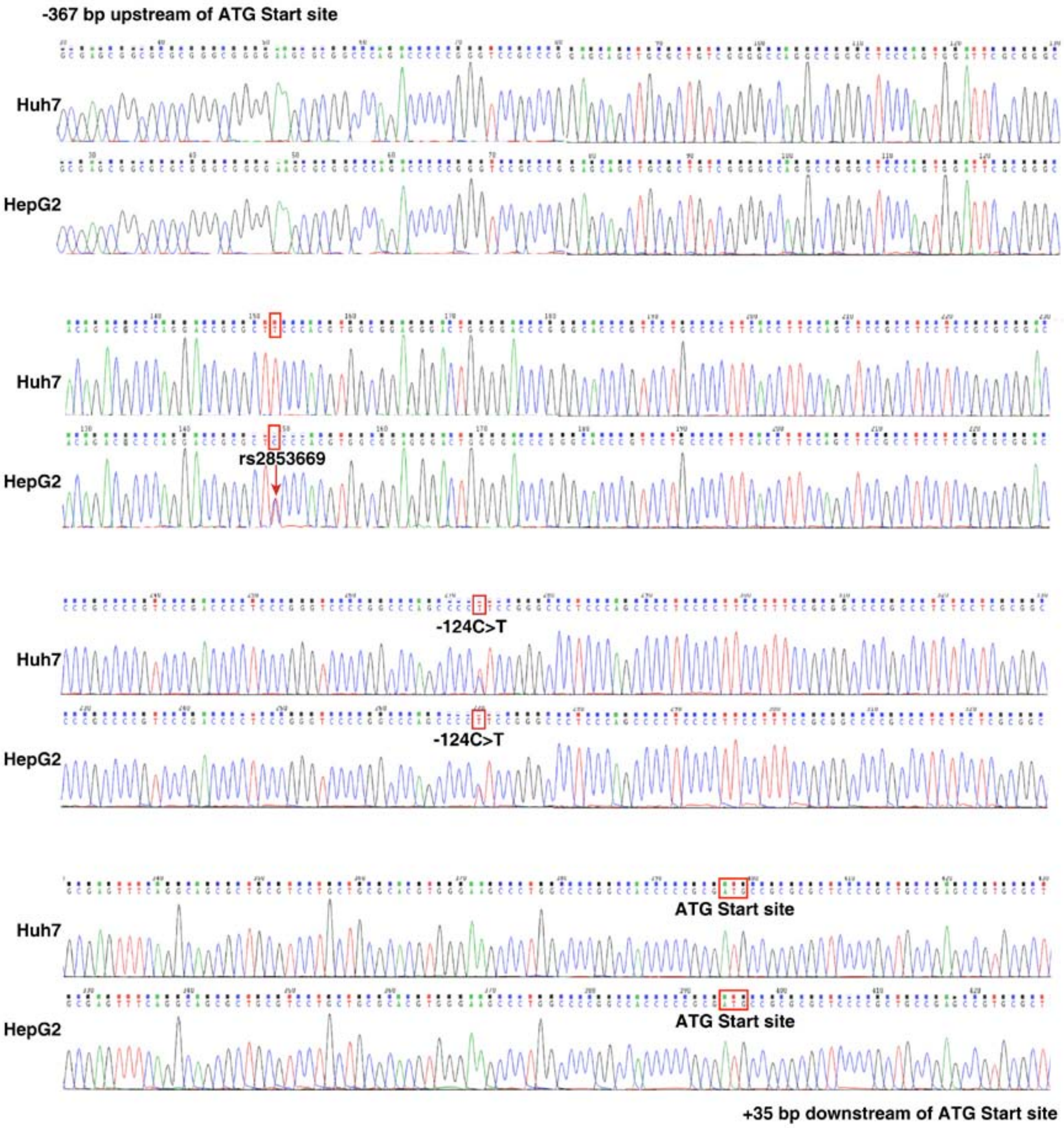

Supplementary Figure S3: *TERT* promoter sequences (from -367 bp upstream of ATG to +35 bp downstream of ATG) from the HCC cell lines Huh7 and HepG2. The Huh7 cells lack the rs2853669 variant at the -245 bp upstream of ATG. The HepG2 cells include the rs2853669 variant. Except for the variant, the Huh7 and HepG2 sequences are the same from -367 bp upstream of human the *TERT* gene ATG to +35 bp downstream of the human *TERT* gene ATG, including the -124C > T mutation.

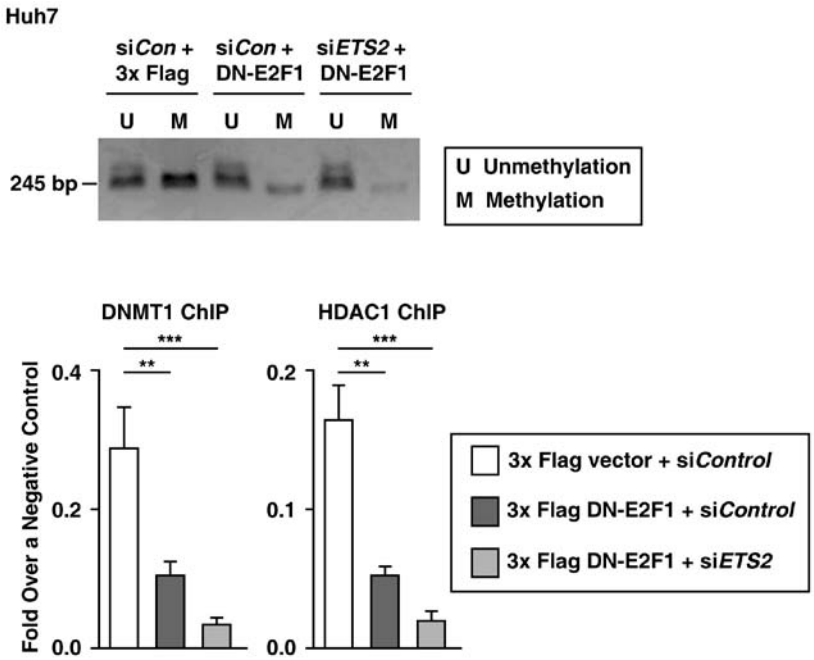

Supplementary Figure S4: MS-PCR using the primer for the *TERT* CpG island MSP targeting site and ChIP experiments using siControl (siCon)- and siETS2-treated Huh7 cells. The data are shown as the mean ± SEM, $^{**}P < 0.01$, $^{***}P < 0.001$.

A

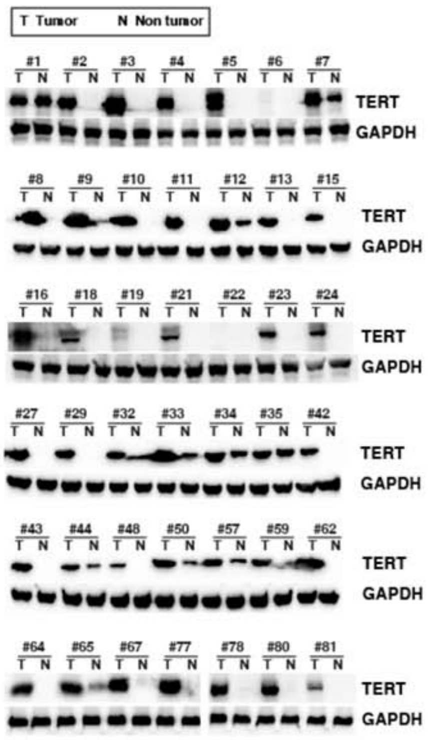

B

HCC case	TERT protein level (ratio of tumor to non tumor)	Methylation level (artificial unit)	HCC case	TERT protein level (ratio of tumor to non tumor)	Methylation level (artificial unit)
#1	1.5	1.2	#27	11.9	0.1
#2	8.8	0.8	#29	30.3	0.9
#3	18.1	1.2	#32	1.9	1.3
#4	10.3	0.8	#33	1.9	0.9
#5	20.5	3.4	#34	1.3	0.4
#6	1.1	0.4	#35	1.0	0.3
#7	1.3	1.2	#42	11.4	0.5
#8	7.3	0.3	#43	39.8	2.8
#9	9.8	0.5	#44	2.7	0.9
#10	21.7	1.5	#48	23.4	1.3
#11	11.4	0.6	#50	3.0	0.2
#12	1.5	0.7	#57	2.2	0.3
#13	20.2	1.6	#59	6.9	0.8
#15	31.0	0.8	#62	21.8	1.1
#16	2.0	0.4	#64	19.1	1.4
#18	5.4	0.3	#65	2.7	0.9
#19	2.6	0.4	#67	11.2	0.7
#21	31.9	1.1	#77	16.7	1.2
#22	1.1	0.3	#78	12.7	1.3
#23	19.7	1.9	#80	19.4	1.0
#24	9.0	0.3	#81	13.4	1.4

Supplementary Figure S5: Association between TERT protein expression levels and *TERT* promoter methylation levels. Immunoblot assay of TERT protein expression in HCC tumor and corresponding non-tumor tissues **A.** and evaluation of the TERT protein expression levels and the *TERT* promoter methylation level **B.** TERT expression was normalized to that of GAPDH.

Supplementary Table S1: Hazard ratios for hepatocellular carcinoma-related death or recurrence

SMH cohort						
	SNP carriers		SNP noncarriers		All	
Patient categories	HR (95% CI)	*P* value	HR (95% CI)	*P* value	HR (95% CI)	*P* value
Death						
Without mutation	1 (Reference)	0.013	1 (Reference)	0.089	1 (Reference)	0.283
With mutation	5.26 (1.42–19.48)		0.34 (0.10–1.18)		1.61 (0.67–3.84)	
Recurrence						
Without mutation	1 (Reference)	0.041	1 (Reference)	0.246	1 (Reference)	0.186
With mutation	5.56 (1.08–28.75)		0.48 (0.14–1.65)		1.94 (0.73–5.17)	

KU cohort						
	SNP carriers		SNP noncarriers		All	
Patient categories	HR (95% CI)	*P* value	HR (95% CI)	*P* value	HR (95% CI)	*P* value
Death						
Without mutation	ND	ND	ND	ND	ND	ND
With mutation						
Recurrence						
Without mutation	1 (Reference)	0.030	1 (Reference)	0.843	1 (Reference)	0.633
With mutation	4.66 (1.16–18.8)		0.78 (0.07–8.66)		1.32 (0.42–4.17)	

SNP, single nucleotide polymorphism; HR, hazard ratio; CI, confidence interval; ND, not detectable.

Supplementary Table S2: Frequencies of *TERT* promoter mutations and rs2853669 variant in tumors from hepatocellular carcinoma patients (SMH Cohort)

	Patients	Somatic mutations			SNP rs2853669	
					Noncarriers	Carriers
Patient categories	n (%)	-124C > T or -146C > T, n(%)	-124C > T, n (%)	-146C > T, n (%)	TT, n (%)	TC or CC, n (%)
All	93 (100.0)	50 (53.8)	50 (100.0)	0 (0.0)	39 (41.9)	54 (58.1)
Gender						
Male	65 (69.9)	38 (58.5)	38 (100.0)	0 (0.0)	31 (47.7)	34 (52.3)
Female	28 (30.1)	12 (42.9)	12 (100.0)	0 (0.0)	8 (28.6)	20 (71.4)
Age, years						
≤ 53	44 (47.3)	27 (61.4)	27 (100.0)	0 (0.0)	20 (45.5)	24 (54.5)
> 53	49 (52.7)	23 (46.9)	23 (100.0)	0 (0.0)	19 (38.8)	30 (61.2)
HCC grade						
Grade I	11 (11.8)	3 (27.3)	3 (100.0)	0 (0.0)	2 (18.2)	9 (81.8)
Grade II	56 (60.2)	33 (58.9)	33 (100.0)	0 (0.0)	30 (53.6)	26 (46.4)
Grade III	26 (28.0)	14 (53.8)	14 (100.0)	0 (0.0)	7 (26.9)	19 (73.1)
AFP, ng/mL						
≤ 15.16	46 (49.5)	24 (52.2)	24 (100.0)	0 (0.0)	17 (37.0)	29 (63.0)
> 15.16	47 (50.5)	26 (55.3)	26 (100.0)	0 (0.0)	22 (46.8)	25 (53.2)
RTL						
Shorter	46 (49.5)	20 (43.5)	20 (100.0)	0 (0.0)	17 (37.0)	29 (63.0)
Longer	47 (50.5)	30 (63.8)	30 (100.0)	0 (0.0)	22 (46.8)	25 (53.2)
Death						
Yes	22 (23.7)	14 (63.6)	14 (100.0)	0 (0.0)	10 (45.5)	12 (54.5)
No	71 (76.3)	35 (49.3)	35 (100.0)	0 (0.0)	29 (40.8)	42 (59.2)
Recurrence						
Yes	18 (19.4)	12 (66.7)	12 (100.0)	0 (0.0)	11 (61.1)	7 (38.9)
No	63 (67.7)	31 (49.2)	31 (100.0)	0 (0.0)	25 (39.7)	38 (60.3)
Missing	12 (12.9)	7 (58.3)	7 (100.0)	0 (0.0)	3 (25.0)	9 (75.0)

SNP, single nucleotide polymorphism; AFP, alpha-fetoprotein; RTL, relative telomere length.

Supplementary Table S3: Frequencies of *TERT* promoter mutations and rs2853669 variant in tumors from hepatocellular carcinoma patients (KU Cohort)

	Patients	Somatic mutations			SNP rs2853669	
					Noncarriers	Carriers
**Patient categories**	**n (%)**	**-124C > T or -146C > T, n (%)**	**-124C > T, n (%)**	**-146C > T, n (%)**	**TT, n (%)**	**TC or CC, n (%)**
All	72 (100.0)	28 (38.9)	27 (96.4)	1 (3.6)	31 (43.1)	41 (56.9)
Gender						
Male	65 (90.3)	27 (41.5)	26 (96.3)	1 (3.7)	29 (44.6)	36 (55.4)
Female	7 (9.7)	1 (14.3)	1 (100.0)	0 (0.0)	2 (28.6)	5 (71.4)
Age, years						
≤ 56	32 (44.4)	10 (31.3)	10 (100.0)	0 (0.0)	13 (40.6)	19 (59.4)
> 56	40 (55.6)	18 (45.0)	17 (94.4)	1 (5.6)	18 (45.0)	22 (55.0)
HCC grade						
Grade I	5 (6.9)	3 (60.0)	3 (100.0)	0 (0.0)	4 (80.0)	1 (20.0)
Grade II	34 (47.2)	13 (38.2)	13 (100.0)	0 (0.0)	12 (35.3)	22 (64.7)
Grade III	33 (45.8)	12 (36.4)	11 (91.7)	1 (8.3)	15 (45.5)	18 (54.5)
AFP, ng/mL						
≤ 15.5	31 (43.1)	13 (41.9)	13 (100.0)	0 (0.0)	14 (45.2)	17 (54.8)
> 15.5	33 (45.8)	10 (30.3)	9 (90.0)	1 (10.0)	11 (33.3)	22 (66.7)
Missing	8 (11.1)	5 (62.5)	5 (100.0)	0 (0.0)	6 (75.0)	2 (25.0)
TERT mRNA level						
Lower	36 (50.0)	13 (36.1)	13 (100.0)	0 (0.0)	18 (50.0)	18 (50.0)
Higher	36 (50.0)	15 (41.7)	14 (93.3)	1 (6.7)	13 (36.1)	23 (63.9)
Recurrence						
Yes	12 (16.7)	5 (41.7)	4 (80.0)	1 (20.0)	3 (25.0)	9 (75.0)
No	56 (77.8)	20 (35.7)	20 (100.0)	0 (0.0)	24 (42.9)	32 (57.1)
Missing	4 (5.6)	3 (75.0)	3 (100.0)	0 (0.0)	4 (100.0)	0 (0.0)

SNP, single nucleotide polymorphism; AFP, alpha-fetoprotein.
